# Effects of Crosstalk Caused by Finite Extinction Ratio in Optical Time-Division Demultiplexer for PADC

**DOI:** 10.3390/mi17101188

**Published:** 2026-10-13

**Authors:** Xin Chen, Meicheng Fu, Yi Zhang, Hongyu Zhang, Hongtao Yu, Yi Zhao, Wenjun Yi, Xiujian Li

**Affiliations:** College of Science, National University of Defense Technology, Changsha 410073, China; dfcx96@163.com (X.C.); yizhangan1er@163.com (Y.Z.); zhanghya1006@163.com (H.Z.); yuhongtao@nudt.edu.cn (H.Y.); zhaoyi21@nudt.edu.cn (Y.Z.); yiwenjun@nudt.edu.cn (W.Y.)

**Keywords:** microwave photonics, extinction ratio, photonic analog-to-digital converters, time-division demultiplexer

## Abstract

Optical time-division demultiplexing photonic analog-to-digital converters (PADCs) represent a compelling paradigm for breaking the bandwidth–resolution trade-off of conventional electronic ADCs, yet inter-channel crosstalk induced by the finite extinction ratio of demultiplexing modulators imposes critical limitations on channel response flatness. Prior investigations have generally ascribed periodic response ripples exclusively to backend bandwidths narrower than half the total sampling rate, while the distortion behavior under wider bandwidths remains inadequately clarified. In this numerical work, we establish a full-link numerical model of a two-channel time-division demultiplexing PADC operating at a total sampling rate of 10 GS/s and systematically characterize crosstalk-induced frequency responses under four representative backend bandwidths (2.5, 5, 7.5, and 10 GHz) with an extinction ratio of 10 dB. Results demonstrate that prominent 10 GHz periodic ripples persist in the channel response even when the backend bandwidth exceeds half the sampling rate. The underlying spectral mechanism is elucidated: demultiplexed comb lines follow a 10 GHz periodic strength distribution, and sampling-generated sidebands inherit the amplitude of their parent comb teeth; the superposition of strong and weak sidebands within the passband governs the ripple magnitude. These findings furnish fundamental insights into crosstalk physics and deliver actionable guidance for device selection and system-level optimization in high-performance PADC implementations.

## 1. Introduction

Compared with continuously-varying analog signals, digital signals encode information in discrete states and deliver better transmission and processing performance. Although modern electronic analog-to-digital converters (EADCs) achieve improved bandwidth and resolution utilizing time-interleaving and digital calibration, device non-idealities including clock jitter, channel mismatch and analog-circuit nonlinearity impose an intractable inherent trade-off among energy efficiency, bandwidth and resolution [1,2,3,4], forming critical bottlenecks that limit conventional EADCs for next-generation broadband RF receivers [5]. Photonic analog-to-digital converters (PADCs) shift core digitization processes into the optical domain [6,7,8,9,10,11,12]. Leveraging the intrinsic ultrafast response and enormous bandwidth of photons, they bypass the speed barriers of electronic hardware and fundamentally alleviate electrical imperfections such as clock jitter and channel mismatch. Moreover, recent advances in integrated photonic analog preprocessing have opened promising prospects for high-performance broadband microwave photonic systems [13,14,15]. The PADC is the pivotal interface between optical-domain signal conditioning and electronic digitization, which is inherently complementary to these emerging preprocessing technologies. The synergy between advanced optical preprocessing schemes and high-performance PADC frontends is anticipated to drive further breakthroughs in ultra-wideband microwave photonic applications.

As the scheme most extensively investigated, the optical-sampling electrical-quantization PADC performs sampling in the optical domain while quantization is performed by the backend EADC [16]. However, the high-speed pulse train from optical sampling is incompatible with the rate limit of the backend EADC. Optical demultiplexing is thus required to distribute the serial optical stream into multiple parallel low-speed channels for subsequent photoelectric conversion and electrical quantization.

On-chip soliton pulse sources and high-speed actively mode-locked lasers directly generate optical pulses at tens-of-gigahertz repetition rates with excellent inter-pulse consistency, low timing jitter and phase noise, acting as well-suited sampling sources for optical-sampling electrical-quantization PADCs [17,18,19,20]. They eliminate the need for sophisticated external pulse time- and wavelength-interleaving modules, which simplifies the optical sampling frontend architecture and facilitates chip-scale integration of PADC systems. High-speed optical switches are used to demultiplex the high-rate optical-sampling pulse trains, which periodically select pulses from the high-speed serial stream and distribute them to distinct output ports in a time-interleaved manner [19]. The original serial high-speed pulses are split into multiple parallel low-speed optical channels with a greatly reduced per-channel repetition rate, matching the speed limit of backend electronic ADCs. Juodawlkis et al. proposed using the cascaded dual-output Mach–Zehnder Interferometer (MZI) modulators for an optical time-division demultiplexer [21], and Williamson et al. analyzed the demultiplexing-induced errors, proving that demultiplexer crosstalk and detector-circuit memory effects produce time-interleaved spurs and degrade the spurious-free dynamic range (SFDR) [22]. Recently, Wang Cheng et al. further distinguished inter-channel crosstalk and intra-channel crosstalk caused by insufficient backend bandwidth. Specially, when a backend bandwidth is less than half of the overall sampling rate, inter-channel crosstalk will cause a periodical variation in the corresponding channel magnitude response in mismatch-free time-interleaved photonic analog-to-digital converters (TIPADCs), with the period being the overall sampling rate [23]. In contrast, our work reveals a distinct physical mechanism: the finite extinction ratio of the dual-output MZI demultiplexer leads to incomplete time-division gating and inter-channel leakage, which imposes a 10 GHz periodic amplitude modulation on the demultiplexed spectral comb.

In this paper, we further investigate inter-channel crosstalk introduced by the finite extinction ratio of the dual-output MZI modulator based an optical time-division demultiplexer theoretically and numerically. The results show that such crosstalk can generate periodic fluctuations in the channel frequency response of PADCs, even if the backend electrical bandwidth exceeds half of the total system sampling rate. A simulation model of a two-channel time-division demultiplexer PADC is established, with a per-channel sampling rate of 5 GS/s and a total system sampling rate of 10 GS/s. Simulation results demonstrate that distinct periodic fluctuations still occur in the channel frequency response when the backend electrical bandwidth is set to 7.5 GHz. These findings may provide theoretical guidance for device selection and system-level optimization during PADC system implementation.

## 2. Theory of Time-Division Demultiplexing PADC

**Figure 1:**
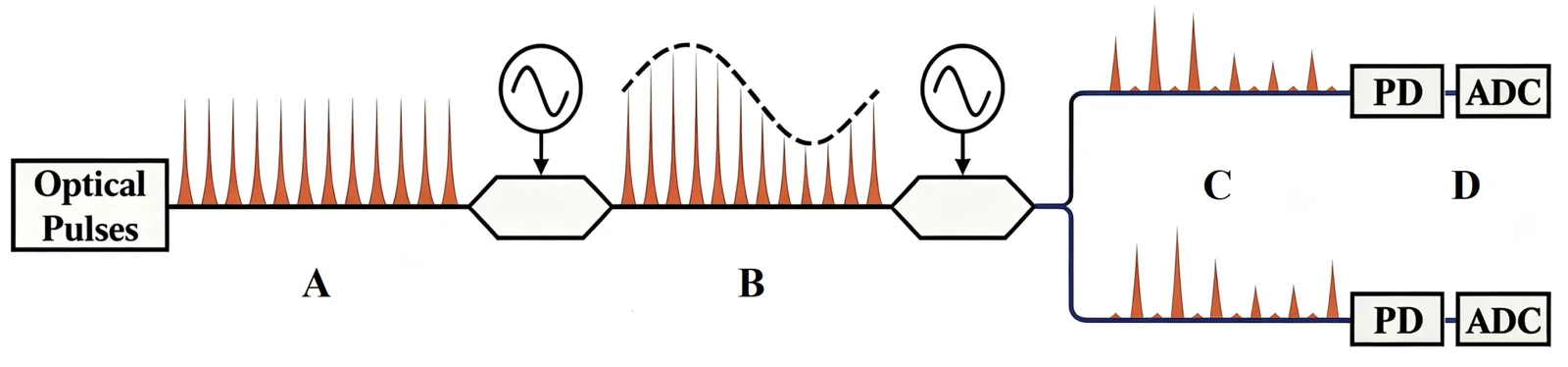
Schematic of two-channel time-division demultiplexing PADC based on dual-output MZI modulator. A–D denote the signal states at the corresponding nodes along the optical path. Device names: PD, photodetector; ADC, analog-to-digital converter.

Figure 1 shows the schematic diagram of a typical time-division demultiplexing PADC. The low-jitter ultrashort optical pulses act as optical sampling clocks. Radio-frequency signals are applied to an electro-optic modulator, where optical pulses sample analog radio-frequency waveforms. The sampled optical pulses are time-division demultiplexed by a dual-output MZI modulator and distributed into two low-speed output channels. They are then converted into electrical signals by photodetectors (PDs) and quantized by electronic ADCs.

**Figure 2:**
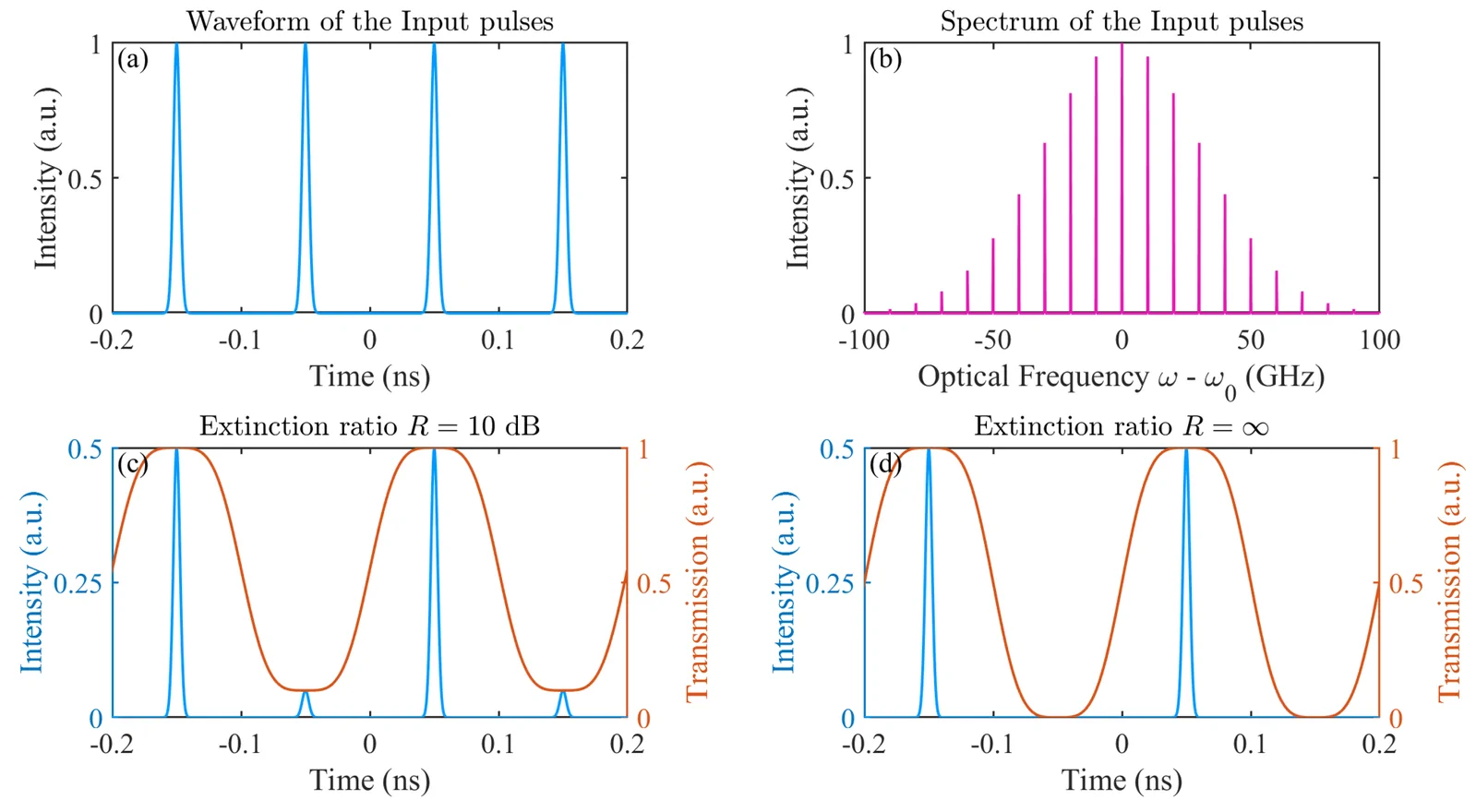
Time-domain waveform and spectrum of input pulses, together with temporal transmission responses of dual-output MZI modulator. Input pulses follow a Gaussian intensity profile with a 6 ps pulse width. (**a**) Waveform of input optical pulses; (**b**) optical spectrum of input pulses; (**c**) modulator transmission characteristic and demultiplexed pulses at extinction ratio R=10dB; (**d**) ideal modulator transmission characteristic and demultiplexed pulses with infinite extinction ratio R=∞. No RF power is applied to the sampling modulator for (**c**,**d**).

Figure 2 illustrates the time-domain waveforms and optical spectrum of input sampling pulses, together with the temporal transmission characteristic of the dual-output MZI modulator under varying extinction ratios. The extinction ratio R (in dB), which characterizes the on–off isolation performance of modulator ports, is defined as $R=10\log_{10}\left(P_{\mathrm{on}}/P_{\mathrm{off}}\right)$, where $P_{\mathrm{on}}$ and $P_{\mathrm{off}}$ denote the maximum on-state output optical power and the minimum off-state output optical power for a single modulator port, respectively. Figure 2a presents the time-domain waveform of input ultrashort optical pulses, while Figure 2b depicts their corresponding optical spectrum, where the input optical pulses adopt a Gaussian intensity profile with a pulse width of 6 ps. In Figure 2c,d, the blue curves represent pulse outputs after demultiplexing by the dual-output modulator, for which no RF driving power is exerted on the sampling modulator. The peak intensity of these blue curves reaches 0.5, a value originating from the quadrature-biased operating point of the sampling modulator, at which the output optical power falls to half of the input power in the absence of RF excitation. Figure 2c corresponds to a finite extinction ratio of R=10dB, where residual optical leakage persists at the off-state and constitutes the physical origin of inter-channel crosstalk. By contrast, Figure 2d describes the ideal scenario with an infinite extinction ratio R=∞, whereby complete suppression of off-state optical power is achieved without any leakage light.

To quantitatively analyze the inter-channel crosstalk induced by the finite extinction ratio of the dual-output MZI demultiplexer, we establish a mathematical model for pulse transmission, where the Gaussian input pulse source, sampling modulator, non-ideal on–off transmission characteristic and limited bandwidth of the backend electronics are all taken into consideration. We begin our model with the pulsed optical source at point A in Figure 1, and the periodic pulse train is denoted as $P_{in}\left(t\right)$. The power envelope of each individual pulse is given by $P_{p}p\left(t\right)$, where $P_{p}$ is the peak pulse power and $\tilde{p}\left(f\right)$ is the Fourier transform of the normalized envelope function p(t). The pulse train can be regarded as a periodic replication of the single-pulse envelope with repetition period $T_{r}$. From Fourier series theory for periodic signals, the optical spectrum consists of discrete spectral lines located at $f=nf_{r}$, with repetition frequency $f_{r}=1/T_{r}$, whose envelope is determined by the Fourier transform of a single pulse. A larger peak power $P_{p}$ and shorter repetition period $T_{r}$ yield a higher average power and higher repetition rate, thus increasing the magnitude of the spectral envelope [24]. The complete time-frequency description of the pulsed source is summarized in Equation (1), as follows:

(1) $$\left\{\begin{array}{c} P_{in}\left(t\right)=P_{p}\sum_{n=-\infty }^{+\infty }p\left(t-nT_{r}\right)\\ {\tilde{P_{in}}}_{(}f)=\sum_{n=-\infty }^{+\infty }\frac{P_{p}}{T_{r}}\tilde{p}\left(nf_{r}\right)\;\delta \left(f-nf_{r}\right) \end{array}\right.$$

Following the pulsed-source characterization at point A, we derive the optical signal after the sampling MZI modulator, corresponding to point B in Figure 1. Under small-signal radio-frequency (RF) driving conditions, $V_{\mathrm{rf}}\left(t\right)$ is the applied RF voltage, and $h_{\mathrm{samp}}\left(t\right)$ denotes the electrical impulse response of the sampling modulator. The actual electrical drive signal experienced by the modulator is the temporal convolution $V_{\mathrm{rf}}\left(t\right)*h_{\mathrm{samp}}\left(t\right)$. $V_{\pi }\left(f\right)$ represents the frequency-dependent half-wave voltage. The output optical power is obtained by multiplying the input pulsed-source power by the Mach–Zehnder modulator (MZM) intensity transfer function. Fourier transformation combined with the convolution theorem yields the output spectrum at point B. The time- and frequency-domain expressions are given by Equation (2):

(2) $$\left\{\begin{array}{c} P_{\mathrm{samp}}\left(t\right)=\left[P_{p}\sum_{n=-\infty }^{+\infty }p\left(t-nT_{r}\right)\right]\frac{1+V_{\mathrm{rf}}\left(t\right)*h_{\mathrm{samp}}\left(t\right)}{2}\\ {\tilde{P}}_{\mathrm{samp}}\left(f\right)=\frac{\left[\sum_{n=-\infty }^{+\infty }\frac{P_{p}}{T_{r}}\;\tilde{p}\left(nf_{r}\right)\;\delta \left(f-nf_{r}\right)\right]}{2}\;+\;\frac{\left[\sum_{n=-\infty }^{+\infty }\frac{P_{p}}{T_{r}}\;\tilde{p}\left(nf_{r}\right)\;\delta \left(f-nf_{r}\right)\right]*\frac{\pi {\tilde{V}}_{\mathrm{rf}}\left(f\right)}{V_{\pi }\left(f\right)}}{2} \end{array}\right.$$

where $P_{\mathrm{samp}}\left(t\right)$ and ${\tilde{P}}_{\mathrm{samp}}\left(f\right)$ are the time-domain output optical power and its Fourier-domain spectrum at point B, respectively. ${\tilde{V}}_{\mathrm{rf}}\left(f\right)$ is the Fourier transform of the RF driving voltage, and the operator ∗ stands for convolution. Parameters $P_{p}$, $T_{r}$, $f_{r}$, p(t) and $\tilde{p}\left(f\right)$ are inherited from the pulsed optical source at point A. We stress that Equation (2) is valid exclusively for small-signal RF modulation: the first term corresponds to the original optical frequency comb of the pulse source, whereas the second convolution term accounts for sidebands induced by electro-optic modulation. The modulated pulse train at point B is then fed into the dual-output MZI demultiplexer with a time-varying transmission function $T_{gate}\left(t\right)$.

After modulation at point B, the modulated pulse train passes through the dual-output MZI demultiplexer, which imposes a time-domain gating action described by the time-dependent demultiplexing transmission function $T_{\mathrm{gate}}\left(t\right)$. The optical pulses after the demultiplexer are obtained by multiplying the MZM output waveform $P_{\mathrm{samp}}\left(t\right)$ with this gate transmission $T_{\mathrm{gate}}\left(t\right)$. The Fourier-domain spectrum follows from the convolution theorem: temporal multiplication by $T_{\mathrm{gate}}\left(t\right)$ corresponds to frequency-domain convolution with its Fourier transform ${\tilde{T}}_{\mathrm{gate}}\left(f\right)$. The time- and frequency-domain expressions for the demultiplexed signal are $P_{\mathrm{demux}}\left(t\right)$ and ${\tilde{P}}_{\mathrm{demux}}\left(f\right)$, as given in Equation (3):

(3) $$\left\{\begin{array}{c} P_{\mathrm{demux}}\left(t\right)=\left[P_{p}\sum_{n=-\infty }^{+\infty }p\left(t-nT_{r}\right)\right]\frac{1+V_{\mathrm{rf}}\left(t\right)*h_{\mathrm{samp}}\left(t\right)}{2}\;T_{\mathrm{gate}}\left(t\right)\\ {\tilde{P}}_{\mathrm{demux}}\left(f\right)={\tilde{P}}_{\mathrm{samp}}\left(f\right)*{\tilde{T}}_{\mathrm{gate}}\left(f\right)=\{\frac{1}{2}\left[\sum_{n=-\infty }^{+\infty }\frac{P_{p}}{T_{r}}\;\tilde{p}\left(nf_{r}\right)\;\delta \left(f-nf_{r}\right)\right]\\ \;+\frac{1}{2}\left[\sum_{n=-\infty }^{+\infty }\frac{P_{p}}{T_{r}}\;\tilde{p}\left(nf_{r}\right)\;\delta \left(f-nf_{r}\right)\right]*\frac{\pi {\tilde{V}}_{\mathrm{rf}}\left(f\right)}{V_{\pi }\left(f\right)}\}*{\tilde{T}}_{\mathrm{gate}}\left(f\right) \end{array}\right.$$

After passing through the time-division demultiplexer with an optical gating function $T_{\mathrm{gate}}\left(t\right)$, the optical signal impinges on the photodetector [25]. All subsequent electrical responses of the PD, transimpedance amplifier, filter, ADC and other backend electronics are aggregated into the combined linear impulse-response $h_{\mathrm{pd}}\left(t\right)$ (frequency-domain transfer function $H_{\mathrm{pd}}\left(f\right)$). Quantization noise and other noise, such as time jitter and RIN, are not included in this model, as we focus on the crosstalk distortion. The full gated optical power waveform is convolved with $h_{\mathrm{pd}}\left(t\right)$ to yield the output photocurrent $i_{\mathrm{pd}}\left(t\right)$. The time- and frequency-domain expressions are $i_{\mathrm{pd}}\left(t\right)$ and ${\tilde{I}}_{\mathrm{pd}}\left(f\right)$, as shown in Equation (4). Note that the photodetector responsivity and backend gain are normalized.

(4) $$\left\{\begin{array}{c} i_{\mathrm{pd}}\left(t\right)=\left\{\left[P_{p}\sum_{n=-\infty }^{+\infty }p\left(t-nT_{r}\right)\right]\frac{1+V_{\mathrm{rf}}\left(t\right)*h_{\mathrm{mzm}}\left(t\right)}{2}\;T_{\mathrm{gate}}\left(t\right)\right\}*h_{\mathrm{pd}}\left(t\right)\\ {\tilde{I}}_{\mathrm{pd}}\left(f\right)=\{\left(\begin{array}{c} \frac{1}{2}\left[\sum_{n=-\infty }^{+\infty }\frac{P_{p}}{T_{r}}\;\tilde{p}\left(nf_{r}\right)\;\delta \left(f-nf_{r}\right)\right]\\ \;+\frac{1}{2}\left[\sum_{n=-\infty }^{+\infty }\frac{P_{p}}{T_{r}}\;\tilde{p}\left(nf_{r}\right)\;\delta \left(f-nf_{r}\right)\right]*\frac{\pi {\tilde{V}}_{\mathrm{rf}}\left(f\right)}{V_{\pi }\left(f\right)} \end{array}\right)*{\tilde{T}}_{\mathrm{gate}}\left(f\right)\}\cdot H_{\mathrm{pd}}\left(f\right) \end{array}\right.$$

## 3. Results and Mechanism Analysis

Numerical simulations based on the aforementioned theoretical model are carried out to investigate the channel frequency-response characteristics of the PADC under varied electronic backend bandwidths (one-sided −3 dB electrical bandwidth) and time-demultiplexing extinction ratios. In order to focus on the core mechanism of extinction-ratio-induced crosstalk, our numerical model adopts several idealized assumptions. For instance, an ideal rectangular backend filter is assumed, while realistic analog filters exhibit gradual roll-off characteristics, which would slightly smooth the ripple edges and reduce the peak-to-valley amplitude. In addition, perfect timing synchronization and zero timing jitter are assumed. Small-signal modulation is also assumed; however, for large RF drive levels, nonlinear modulation effects would generate inter-modulation products and increase spectral distortion, reducing the effective dynamic range. Additional non-ideal effects including MZM bias drift, optical insertion loss, RF frequency response roll-off, detector thermal noise, shot noise, relative intensity noise (RIN), ADC quantization noise, finite ADC aperture, and channel mismatch are not included in the current model.

**Figure 3:**
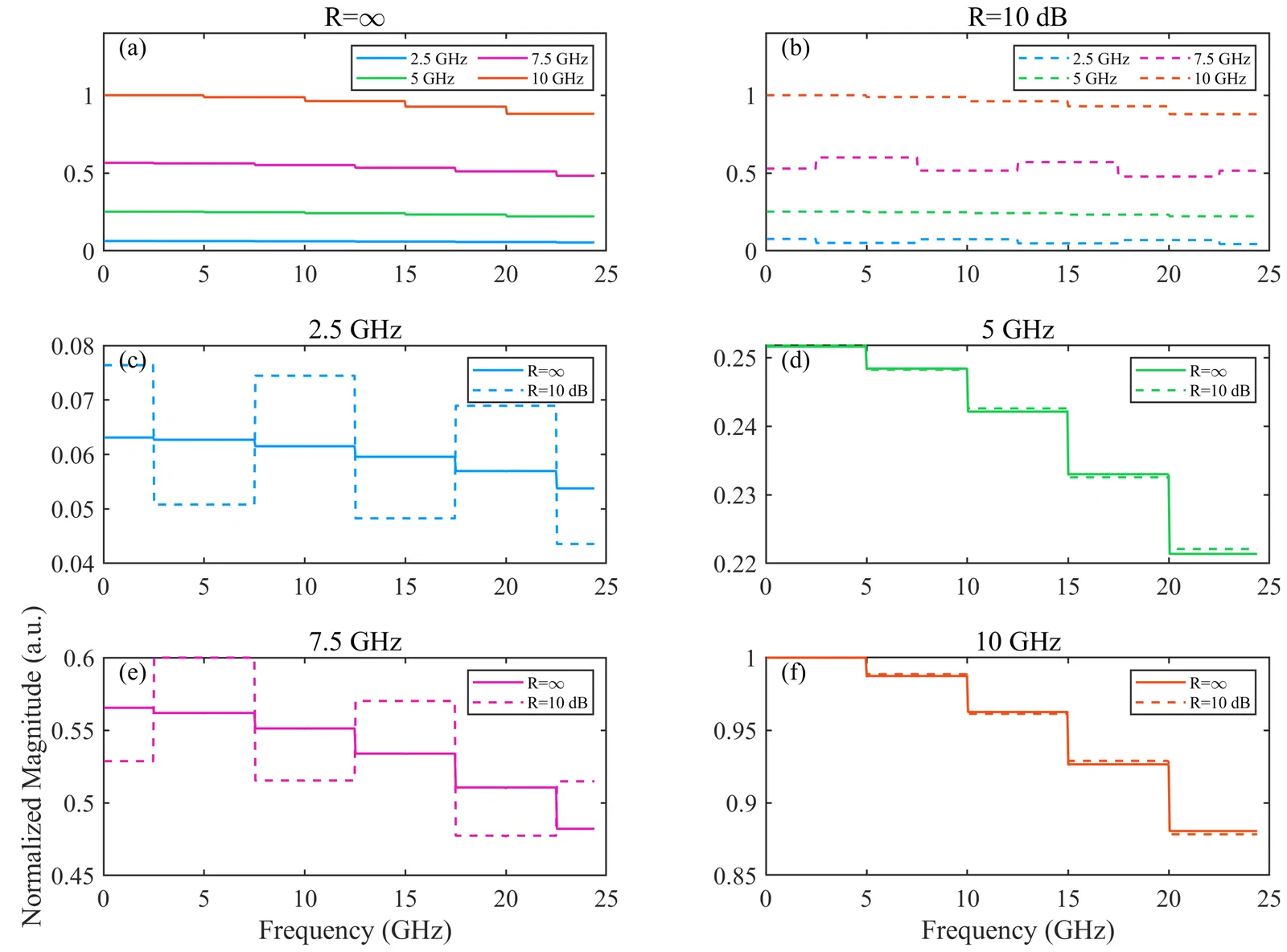
Simulated channel frequency responses under different electronic backend bandwidths and time-demultiplexing extinction ratios. Gaussian-shaped sampling pulses with a FWHM of 6 ps and a pulse repetition rate of 10 GHz are used in the simulation. All curves are normalized against the maximum magnitude of the curve with 10 GHz backend bandwidth in (**a**). (**a**) Results for ideal infinite extinction ratio R=∞. (**b**) Results for finite extinction ratio R=10 dB. The channel-response magnitude increases with growing backend bandwidth in both panels, and obvious ripples caused by crosstalk from a non-ideal time-division demultiplexer are observed in panel (**b**). (**c**–**f**) Overlaid channel responses of R=∞ (solid lines) and R=10 dB (dashed lines) for electronic backend bandwidths of 2.5 GHz, 5 GHz, 7.5 GHz and 10 GHz, respectively. Distinct periodic ripples with a period of 10 GHz appear for R=10 dB at 2.5 GHz and 7.5 GHz backend bandwidths, while marginal differences exist between two extinction-ratio cases at 5 GHz and 10 GHz.

As illustrated in Figure 3, Gaussian-shaped incident sampling pulses are adopted in the simulation, with a full-width-at-half-maximum (FWHM) of 6 ps and a pulse repetition rate of 10 GHz, which means a time-division demultiplexing PADC with a total sampling rate of 10 GS/s. All numerical simulations in this work are implemented in MATLAB R2019a with custom-built scripts for pulse generation, electro-optic modulation, time-division demultiplexing, and spectral analysis. The simulation is performed in the time domain with a total duration of 409.6 ns, corresponding to 4096 input optical pulses at a 10 GHz repetition rate. The point in the time domain is 4,194,304, corresponding to a time resolution of $9.7656\times 10^{-5}$ ns, which is sufficiently small to resolve the 6 ps Gaussian pulses. Frequency-domain analysis is performed via a 4,194,304-point ($2^{22}$) fast Fourier transform (FFT), yielding a frequency resolution of approximately 2.44 MHz. And the central wavelength of the optical pulses is 1550 nm. To validate the accuracy of our numerical model, we first reproduce the classic inter-channel crosstalk scenario investigated by Wang et al. [23], where the backend bandwidth is narrower than half the total sampling rate. Simulation results show that for a 2.5 GHz backend bandwidth, the channel frequency response exhibits clear periodic ripples with a period of 10 GHz, which is consistent with the conclusion in prior work. This consistency verifies that our modeling framework accurately captures the crosstalk behavior induced by the finite extinction ratio in time-division demultiplexers, and provides a reliable basis for further investigation into wider bandwidth regimes. This work adopts a bandpass undersampling architecture, which is standard in high-speed PADC systems. The per-channel ADC sampling rate is $f_{\mathrm{adc}}=f_{s}/2=5\;\mathrm{GS}/s$, with a first Nyquist zone of [0, 2.5 GHz]. An input RF frequency $f_{\mathrm{in}}$ in the m-th Nyquist zone maps to the baseband alias frequency through $f_{\mathrm{alias}}=\left|f_{\mathrm{in}}-mf_{\mathrm{adc}}\right|$, where m is the Nyquist zone index. All curves are normalized against the maximum magnitude of the 10 GHz backend-bandwidth curve in Figure 3a, which enables valid amplitude comparison among cases with different extinction ratios. Figure 3a presents the ideal case of an infinite time-demultiplexing extinction ratio R=∞, while Figure 3b corresponds to the practical condition R=10 dB. The channel-response magnitude rises with broader backend bandwidth in both cases. The curves in Figure 3a remain smooth and oscillation-free, whereas prominent ripples emerge in Figure 3b, originating from channel crosstalk caused by signal leakage of optical gating windows. To compare system performances under identical backend bandwidth, Figure 3c–f overlay channel responses for R=∞ (solid lines) and R=10 dB (dashed lines) for electronic backend bandwidths of 2.5 GHz, 5 GHz, 7.5 GHz and 10 GHz, respectively. Without loss of generality, the electronic backend is assumed to have an ideal rectangular filtering response with flat in-band gain and perfect out-of-band rejection in this work. For R=10 dB, severe periodic ripples with a period of 10 GHz are captured at approximately 3.2 dB for the 2.5 GHz bandwidth, and 1.3 dB for the 7.5 GHz bandwidth. By contrast, negligible deviation is observed between the two extinction-ratio cases at bandwidths of 5 GHz and 10 GHz.

These observations demonstrate that crosstalk distortion induced by a finite extinction ratio is strongly frequency-selective and tightly dependent on backend bandwidth. Minor discrepancies exist between R=10 dB and R=∞ when the backend bandwidth equals half of the total sampling rate (5 GHz) or the full sampling rate (10 GHz). Nevertheless, pronounced periodic ripples, which deviate substantially from the ideal case, will be triggered under a finite extinction ratio if the backend bandwidth falls below half the sampling rate (2.5 GHz) or reaches three quarters of the sampling rate (7.5 GHz). Simulation results demonstrate that distinct periodic fluctuations still occur in the channel frequency response when the backend electrical bandwidth is even greater than half of the total sampling rate.

**Figure 4:**
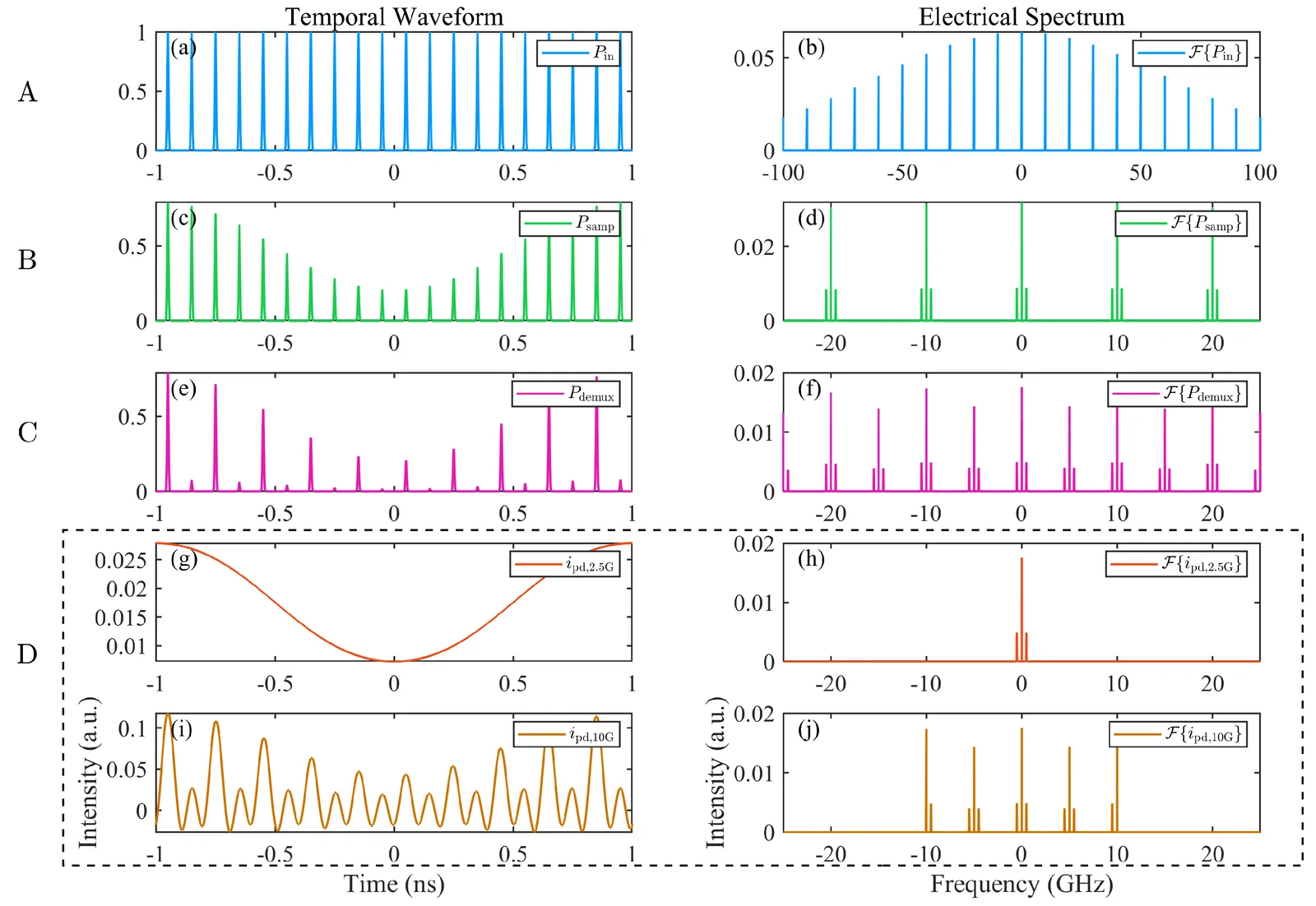
Simulated temporal intensity waveforms and corresponding electrical spectra at key nodes A–D of the PADC system under a finite time-demultiplexing extinction ratio of R=10 dB, where a 500 MHz radio-frequency signal is used as the input to be sampled. All right panels show the Fourier transform of the intensity envelope (electrical spectrum). (**a**,**b**) Temporal waveform and electrical spectrum of input sampling pulse intensity $P_{\mathrm{in}}$ at node A. (**c**,**d**) Temporal waveform and electrical spectrum of sampled pulse intensity $P_{\mathrm{samp}}$ at node B after the sampling modulator. (**e**,**f**) Temporal waveform and electrical spectrum of $P_{\mathrm{demux}}$ at node C after time demultiplexing. Region D (dashed box) shows photocurrent outputs after photodetection for different backend bandwidths: (**g**,**h**) 2.5 GHz backend bandwidth; (**i**,**j**) 10 GHz backend bandwidth.

To intuitively reveal the physical origin of the aforementioned periodic ripples in the channel frequency response from both time and frequency domains, Figure 4 further presents temporal intensity waveforms and corresponding electrical spectra at four key nodes A–D along the time-demultiplexing PADC chain, depicting the complete signal evolution through the system, where the RF frequency applied in the sampling modulator is 500 MHz. The time-demultiplexing stage in this set of simulations is operated under a finite extinction ratio of R=10 dB, and all right-hand panels show the Fourier transform of the intensity envelope, i.e., the electrical spectrum.

Node A corresponds to the input Gaussian sampling pulses, whose temporal intensity envelope $P_{\mathrm{in}}$ is plotted in Figure 4a with a repetition rate of 10 GHz; its electrical spectrum in Figure 4b exhibits comb-like line spectra with a spacing of 10 GHz, determined by the pulse repetition rate. After passing through the sampling modulator, the signal arrives at node B as the sampled optical pulse intensity train $P_{\mathrm{samp}}$ (Figure 4c), whose envelope carries radio-frequency information, and sidebands emerge on the original line spectra in Figure 4d. Node C gives the output $P_{\mathrm{demux}}$ after dual-output MZI time demultiplexing (Figure 4e). Due to incomplete time demultiplexing under the finite extinction ratio, signal leakage and crosstalk exist in the gating window, and residual small pulse components accompany the high-speed optical pulses redistributed into parallel channels. This effect is directly manifested in the electrical spectrum: significantly suppressed power appears at frequency points such as 5 GHz and 15 GHz in Figure 4f. Such selective power attenuation at specific spectral components is the fundamental cause of the 10 GHz periodic ripples observed in the channel frequency response of Figure 3.

Region D presents photocurrents after photodetection under different backend electrical bandwidths. On one hand, a larger backend electrical bandwidth preserves more high-frequency spectral components and yields higher overall output signal intensity, which gives rise to a larger channel frequency-response magnitude, fully consistent with the rising trend in response magnitude with bandwidth in Figure 3. On the other hand, when the backend electrical bandwidth is narrow, residual small pulses induced by imperfect time demultiplexing will undergo temporal broadening after photodetection due to the bandwidth limitation, which in turn causes mutual interference with the main pulses and further aggravates channel-response distortion. For a 2.5 GHz backend bandwidth, $i_{\mathrm{pd},2.5G}$ yields a slowly varying temporal envelope (Figure 4g) with only low-frequency components surviving in its spectrum (Figure 4h); when the backend bandwidth increases to 10 GHz, $i_{\mathrm{pd},10G}$ resolves faster signal variations with obvious temporal oscillations (Figure 4i), and higher-frequency components are preserved in its spectrum (Figure 4j).

From the perspective of signal evolution, these results confirm that time demultiplexing rearranges pulse temporal positions, and incomplete demultiplexing with leakage components induced by a finite extinction ratio acts as the source of crosstalk distortion. Meanwhile, the subsequent electronic backend bandwidth not only governs the retention of high-frequency components but also modulates the interference strength of residual crosstalk through the pulse-broadening effect. Their combined effect accounts for the frequency-selective distortion observed in Figure 3.

**Figure 5:**
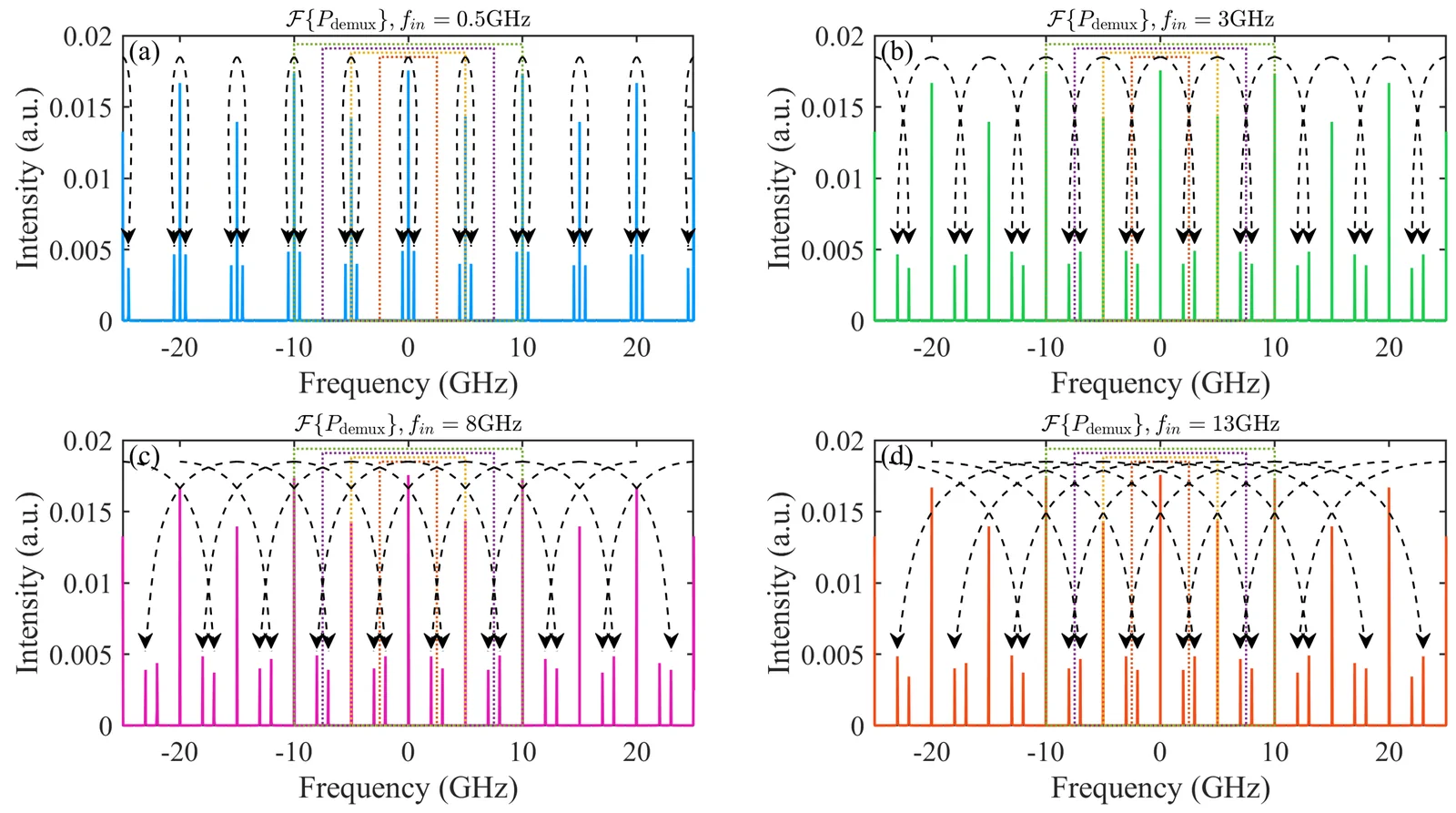
Simulated electrical spectra of the time-demultiplexed output intensity $P_{\mathrm{demux}}$ under different input RF frequencies with an extinction ratio of R=10 dB. (**a**) $f_{\mathrm{in}}=0.5$ GHz; (**b**) $f_{\mathrm{in}}=3$ GHz; (**c**) $f_{\mathrm{in}}=8$ GHz; (**d**) $f_{\mathrm{in}}=13$ GHz. Black dashed lines indicate the correspondence between each main comb tooth and its sampling-generated RF sidebands. Colored dashed boxes from inside to outside correspond to electronic backend bandwidth passbands of ±2.5 GHz, ±5 GHz, ±7.5 GHz and ±10 GHz, respectively. The demultiplexed comb teeth have a 5 GHz spacing, and their amplitudes follow a 10 GHz periodic strength distribution due to the non-ideal finite extinction ratio of time demultiplexing.

To further clarify the spectral origin of the periodic ripples in channel frequency response, comparative simulations are performed on the output spectra after time demultiplexing under different input RF frequencies, as shown in Figure 5. All simulations are conducted under a finite time-demultiplexing extinction ratio of R=10 dB, with input RF frequencies set to 0.5 GHz, 3 GHz, 8 GHz and 13 GHz, respectively. Black dashed lines indicate the correspondence between main comb lines and their sampling-generated RF sidebands: each dashed line starts from a main comb tooth and points to its pair of upper and lower sidebands produced by sampling modulation. Colored dashed boxes from inside to outside correspond to the passbands of electronic backend bandwidths of ±2.5 GHz, ±5 GHz, ±7.5 GHz and ±10 GHz, respectively; only spectral components falling within the corresponding passband can pass through the backend circuit and be acquired.

The demultiplexed spectrum exhibits a 5 GHz spaced comb structure as a consequence of the two-channel time demultiplexing. However, due to the non-ideal effect of time demultiplexing, the comb-tooth amplitudes follow a distinct 10 GHz periodic strength distribution: main comb teeth at integer multiples of 10 GHz, such as 0, ±10 and ±20 GHz, possess higher amplitudes, while those at ±5, ±15 and ±25 GHz are noticeably weaker. During sampling modulation, each main comb tooth acts as a carrier and generates a pair of upper and lower sidebands, whose amplitude directly inherits the original amplitude of its parent comb tooth. The final channel response at a given input frequency is determined by the superposition of all effective spectral components falling within the backend bandwidth passband.

Taking the 2.5 GHz backend bandwidth as an example, the periodic ripple pattern of the channel frequency response shown in Figure 3 can be clearly explained with Figure 5:

- At an input RF frequency of 0.5 GHz (Figure 5a), the ±2.5 GHz passband contains only a pair of sidebands (±0.5 GHz) from the strong main comb tooth at 0 GHz. All effective components originate from the high-amplitude 0 GHz main comb tooth, resulting in high total signal strength and a high channel respons.
- When the input RF frequency rises to 3 GHz (Figure 5b), the sidebands of the 0 GHz main comb tooth fall at ±3 GHz, outside the ±2.5 GHz passband. Only one pair of sidebands exists within the passband: the lower sideband of the +5 GHz main comb tooth (5 GHz − 3 GHz = 2 GHz) and the upper sideband of the −5 GHz main comb tooth (−5 GHz + 3 GHz = −2 GHz). Since both sidebands are derived from the low-amplitude ±5 GHz weak main comb teeth, the overall effective signal strength is significantly reduced, and the channel response decreases accordingly.
- At an input frequency of 8 GHz (Figure 5c), the ±2.5 GHz passband contains a pair of sidebands: the lower sideband of the +10 GHz strong main comb tooth (10 GHz − 8 GHz = 2 GHz) and the upper sideband of the −10 GHz strong main comb tooth (−10 GHz + 8 GHz = −2 GHz). Both sidebands originate from the high-amplitude ±10 GHz main comb teeth, so the effective signal strength recovers and the channel response returns to a high level.
- When the input RF frequency reaches 13 GHz (Figure 5d), the effective sidebands in the ±2.5 GHz passband come from the lower sideband of the +15 GHz weak main comb tooth (15 GHz − 13 GHz = 2 GHz) and the upper sideband of the −15 GHz weak main comb tooth (−15 GHz + 13 GHz = −2 GHz). Both sidebands are derived from the low-amplitude ±15 GHz main comb teeth, leading to significant attenuation of the overall effective power and a trough in the channel response.

Building upon the spectral analysis in Figure 5, we further quantify the composition of effective sidebands within the passband for four representative electronic backend bandwidths (2.5 GHz, 5 GHz, 7.5 GHz, and 10 GHz). The detailed characteristics of the parent main comb teeth and sideband strength for each bandwidth are summarized in Table 1, Table 2, Table 3 and Table 4, respectively.

Table 1 presents the case of a 2.5 GHz backend bandwidth. Since the passband width is narrower than the 5 GHz spacing of the demultiplexed comb teeth, only one pair of sidebands originating from the same group of parent main comb teeth falls within the passband at any input frequency. As the input RF frequency increases, the parent main comb teeth of the effective sidebands switch sequentially among 0, ±5, ±10, ±15 GHz, etc., resulting in a periodic strong–weak alternation in sideband strength with a 10 GHz period. This directly accounts for the most pronounced periodic ripples in the channel frequency response for the 2.5 GHz bandwidth, as observed in Figure 3.

Table 2 shows the results for a 5 GHz backend bandwidth. In this case, the passband covers sidebands from two adjacent groups of main comb teeth simultaneously. In each input frequency interval, the passband consistently contains two strong sidebands and two weak sidebands. The superposition of strong and weak components partially cancels out the strength difference, leading to a notable smoothing of the periodic ripples compared with the 2.5 GHz case.

For the 7.5 GHz backend bandwidth, as listed in Table 3, the passband incorporates sidebands from three orders of main comb teeth. The quantity ratio of strong to weak sidebands varies with the input frequency: intervals such as (2.5, 7.5) GHz and (12.5, 17.5) GHz are dominated by strong sidebands, while intervals like (7.5, 12.5) GHz and (17.5, 22.5) GHz are dominated by weak sidebands. This periodic imbalance in total effective power within the passband directly produces the prominent 10 GHz periodic ripple observed in the channel response.

Table 4 summarizes the sideband characteristics for a 10 GHz backend bandwidth. The effective sidebands simultaneously include four strong sidebands and four weak sidebands across every frequency interval. The contributions from strong and weak sidebands tend to be balanced, which maximally suppresses the periodic ripples. Mathematically, this behavior arises from the amplitude-modulated gating term ${\tilde{T}}_{\mathrm{gate}}\left(f\right)$ in Equation (3). The finite extinction ratio breaks the ideal on–off symmetry of the time-division gate, imposing a 10 GHz periodic amplitude envelope on the 5 GHz spaced demultiplexed comb lines. This term exists for all bandwidth values; it only becomes invisible when strong and weak sidebands are exactly balanced within the passband. It should be noted that this counting method is an intuitive physical explanation rather than a rigorous coherent calculation. The core logic is that a flat channel response requires a flat electrical spectrum. Strict quantitative analysis of the phase relationships between all spectral components would require precise modeling of full optical field evolution, which involves more device parameters and is beyond the scope of this work. All quantitative channel response results in this paper are directly obtained from time-domain numerical simulations.

From an experimental perspective, the predicted periodic channel response ripple originating from the finite extinction ratio of the time-division demultiplexer is readily measurable with standard commercially available optoelectronic components. A 10 GHz mode-locked laser or integrated soliton microcomb source delivering 6 ps optical pulses is widely accessible in microwave photonics laboratories and matches the pulse parameters adopted in this work. Dual-output MZI modulators with an extinction ratio of 10 to 20 dB and a 3 dB bandwidth covering 10 GHz are commercially available, and are fully compatible with the demultiplexer configuration in our numerical model. High-speed photodetectors with analog bandwidths of 10 GHz and above are also standard off-the-shelf components for high-speed optical communication and microwave photonics applications. Since the extinction-ratio-induced ripple is a deterministic effect with a peak-to-valley amplitude of several decibels for a 10 dB extinction ratio, it can be clearly distinguished from thermal noise, shot noise and other stochastic impairments in experimental measurements.

## 4. Conclusions

Building upon a deterministic signal-chain model that captures the complete signal evolution from optical pulse generation, electro-optic sampling, and time-division demultiplexing to photodetection, this work systematically investigates the inter-channel crosstalk induced by the finite extinction ratio of a dual-output MZI demultiplexer in a 10 GS/s photonic analog-to-digital converter (PADC) theoretically and numerically. Contrary to prior crosstalk studies that remark that periodic response ripples emerge exclusively when the backend electronic bandwidth is narrower than half the total sampling rate, our results demonstrate that prominent 10 GHz periodic fluctuations persist in the channel frequency response even at a 7.5 GHz backend bandwidth, which exceeds the half-sampling-rate threshold of 5 GHz.

The physical mechanism underpinning this frequency-selective ripple behavior, which is rigorously elucidated via spectral decomposition, originates from the periodic amplitude modulation of demultiplexed comb lines caused by non-ideal gating leakage. Specifically, the time-division demultiplexer with a finite extinction ratio reshapes the original 10 GHz spaced pulse train into a 5 GHz spaced spectral comb whose line strengths follow a distinct 10 GHz periodic alternation between strong and weak values. As each comb line acts as an optical carrier for sampling modulation, the generated RF sidebands directly inherit the amplitude of their parent main comb teeth, so that the superposition of effective sidebands falling within the backend passband ultimately determines the local magnitude of the channel response. As the backend bandwidth broadens, more comb orders and their associated sidebands are admitted into the passband, whereby the partial cancellation between strong and weak sidebands progressively suppresses ripple amplitude. While the 2.5 GHz bandwidth exhibits the most severe ripples, owing to its alternation between purely strong and purely weak sideband components, the 10 GHz bandwidth achieves nearly flat response characteristics because strong and weak sidebands contribute almost uniformly across all frequency intervals.

Taken together, these findings not only deepen the fundamental understanding of crosstalk-induced distortion in time-demultiplexed PADC systems but also establish a quantitative analytical framework that clarifies the inherent correlation among demultiplexer extinction ratio non-ideality and backend bandwidth configuration. From an engineering perspective, the revealed spectral mechanism and quantitative sideband composition rules provide actionable guidance for device selection, bandwidth matching, and system-level performance optimization in practical PADC implementations.

## Figures and Tables

**Table 1:** Characteristics of effective sidebands at different input frequencies with a 2.5 GHz backend bandwidth.

Input RF Frequency Range	Parent Main Comb Teeth	Effective Sideband Property
(0, 2.5) GHz	0GHz	2 strong sidebands
(2.5, 7.5) GHz	±5GHz	2 weak sidebands
(7.5, 12.5) GHz	±10GHz	2 strong sidebands
(12.5, 17.5) GHz	±15GHz	2 weak sidebands
(17.5, 22.5) GHz	±20GHz	2 strong sidebands
(22.5, 27.5) GHz	±25GHz	2 weak sidebands

**Table 2:** Characteristics of effective sidebands at different input frequencies with a 5 GHz backend bandwidth.

Input RF Frequency Range	Parent Main Comb Teeth	Effective Sideband Property
(0, 5) GHz	0, ±5GHz	2 strong + 2 weak sidebands
(5, 10) GHz	±5, ±10GHz	2 strong + 2 weak sidebands
(10, 15) GHz	±10, ±15GHz	2 strong + 2 weak sidebands
(15, 20) GHz	±15, ±20GHz	2 strong + 2 weak sidebands
(20, 25) GHz	±20, ±25GHz	2 strong + 2 weak sidebands
(25, 30) GHz	±25, ±30GHz	2 strong + 2 weak sidebands

**Table 3:** Characteristics of effective sidebands at different input frequencies with a 7.5 GHz backend bandwidth.

Input RF Frequency Range	Parent Main Comb Teeth	Effective Sideband Property
(0, 2.5) GHz	0, ±5GHz	2 strong + 4 weak sidebands
(2.5, 7.5) GHz	0, ±5, ±10GHz	4 strong + 2 weak sidebands
(7.5, 12.5) GHz	±5, ±10, ±15GHz	2 strong + 4 weak sidebands
(12.5, 17.5) GHz	±10, ±15, ±20GHz	4 strong + 2 weak sidebands
(17.5, 22.5) GHz	±15, ±20, ±25GHz	2 strong + 4 weak sidebands
(22.5, 27.5) GHz	±20, ±25, ±30GHz	4 strong + 2 weak sidebands

**Table 4:** Characteristics of effective sidebands at different input frequencies with a 10 GHz backend bandwidth.

Input RF Frequency Range	Parent Main Comb Teeth	Effective Sideband Property
(0, 5) GHz	0, ±5, ±10GHz	4 strong + 4 weak sidebands
(5, 10) GHz	0, ±5, ±10, ±15GHz	4 strong + 4 weak sidebands
(10, 15) GHz	±5, ±10, ±15, ±20GHz	4 strong + 4 weak sidebands
(15, 20) GHz	±10, ±15, ±20, ±25GHz	4 strong + 4 weak sidebands
(20, 25) GHz	±15, ±20, ±25, ±30GHz	4 strong + 4 weak sidebands
(25, 30) GHz	±20, ±25, ±30, ±35GHz	4 strong + 4 weak sidebands

## Data Availability

The original contributions presented in this study are included in the article. Further inquiries can be directed to the corresponding authors.

## Abbreviations

PADC	Photonic Analog-to-Digital Converter
EADC	Electronic Analog-to-Digital Converter
MZI	Mach–Zehnder Interferometer
SFDR	Spurious-Free Dynamic Range
TIPADC	Time-Interleaved Photonic Analog-to-Digital Converter
RF	Radio Frequency
PD	Photodetector
FWHM	Full Width at Half Maximum
FFT	Fast Fourier Transform
RIN	Relative Intensity Noise
